# Intersectoral action for health equity: a rapid systematic review

**DOI:** 10.1186/1471-2458-13-1056

**Published:** 2013-11-09

**Authors:** Sume Ndumbe-Eyoh, Hannah Moffatt

**Affiliations:** 1 National Collaborating Centre for Determinants of Health, P.O.Box 5000, Antigonish NS B2G 2 W5, Canada

**Keywords:** Intersectoral action, Health equity, Socioeconomic factors, Social determinants of health

## Abstract

**Background:**

Action on the social determinants of health is considered a necessary approach to improving health equity. Most of the social determinants of health lie outside the sphere of the health sector and thus collaboration with governmental and non-governmental sectors outside of health are required to develop policies and programs to improve health equity. Case studies of intersectoral action are available, however there is limited information about the impact of intersectoral action on the social determinants of health and health equity.

**Methods:**

Search and retrieval of literature published between 2001 and 2011 was conducted in 6 databases. A staged screening of titles and abstracts, and later full-text, was conducted by two independent reviewers. Reviewers independently assessed the quality of the articles deemed relevant for inclusion. Data were extracted and synthesized in narrative format for all included studies, conducted by one reviewer and checked by another.

**Results:**

17 articles of varied methodological quality met the inclusion criteria. One systematic review investigating partnership interventions found mixed and limited impacts on health outcomes. Primary studies evaluating the impact of upstream and midstream interventions showed mixed effects. Downstream interventions were generally moderately effective in increasing the availability and use of services by marginalized communities.

**Conclusions:**

The literature evaluating the impact of intersectoral action on health equity is limited. The included studies identified reveal a moderate to no effect on the social determinants of health. The evidence on the impact of intersectoral action on health equity is even more limited. The lack of evidence should not be interpreted as a lack of effect. Rigorous evaluations of intersectoral action are needed to strengthen the evidence base of this public health practice.

## Thumbnail

### What is already known on this topic

•Examples and case studies from Canadian and international settings describing intersectoral action for health equity are available [1-6] however there is limited information about the impact of intersectoral action on the social determinants of health and health equity [3,5,7].

•This review systematically assesses the impact and effectiveness of intersectoral action in public health on the social determinants of health and health equity using literature from a number of countries.

### What this study adds

•The body of literature on intersectoral action as a public health practice for advancing health equity is mixed, revealing moderate to no effect on the social determinants of health. Much of the available literature is descriptive and programs are not rigorously evaluated.

•Creating an interdisciplinary body of knowledge about how to evaluate intersectoral action, along with supporting tools, will help strengthen the evidence base for intersectoral action on health equity and the social determinants of health.

•Collaborations between public health and other sectors show promise in creating supportive environments and enhancing access to services for marginalized populations.

## Background

Health equity, “the absence of unfair and avoidable or remediable differences in health among population groups defined socially, economically, demographically or geographically [8]” is increasing becoming a pressing public health issue globally. Health inequities are health differences that are socially produced, systematic across the population, and unfair [9]. Action on the social determinants of health is considered a key approach to improving health equity. The social determinants of health (SDH) are social and economic factors that influence health. They are “the circumstances in which people are born, grow up, live, work and age, and the systems put in place to deal with illness. These circumstances are in turn shaped by a wider set of forces: economics, social policies, and politics” [8]. Examples of the social determinants of health include income and income distribution, education, social safety networks, employment and working conditions, unemployment and job security, early childhood development, gender, race, food insecurity, housing, social exclusion, access to health services, Aboriginal status, and disability [10].

The most significant social determinants of health lie outside the health sector. As such, action within and between sectors, at the local, regional, provincial, national, and global levels, is needed to influence the social and economic landscape that enables the health and well-being of the population [11]. Intersectoral action recognizes that the social and economic factors influencing the health of the population [10,12] lie outside the sphere of the health sector, falling within the purview of other sectors.

Numerous previous reports have noted the importance of intersectoral action in improving health equity [1-3,13-15]; and intersectoral action has been identified as a public health practice with potential to allow local public health units to address the SDH and reduce health inequities [14].

This expedited systematic review was conducted as part of ongoing work at the National Collaborating Centre for Determinants of Health (NCCDH) that explores research and practice evidence to improve health equity through action on the social determinants of health.

For the purposes of this review, we considered intersectoral interventions, policies and programs, undertaken by the public health sector in collaboration with governmental and non-governmental sectors outside of health. We define the public health sector as organizations and individuals that deliver activities intended to reduce the amount of disease, premature death, and disease-related discomfort and disability in the population.

Four patterns of relationships between sectors can be characterized, information-sharing, cooperation, coordination, and integration [13]. An informative relationship is based on information sharing and exchange between sectors; cooperation refers to the achievement of greater efficiency through optimization of resources for the enforcement or implementation of policies or programs; coordination involves joint work among sectors for greater efficiency and effectiveness, generally the creation and integration of synergistic relationships and shared financing; and integration refers to approaching a new policy or program in conjunction with multiple sectors and requires the synthesis of objectives, administrative processes, resources, responsibilities, and actions. Effective engagement across sectors encourages all sectors to examine how their policy and programs can improve health and health equity.

The aim of our review was to examine the impact and effectiveness of intersectoral action as a public health practice for health equity through action on the SDH. Additional questions of interest were the role of the public health sector in intersectoral action and the tools, mechanisms, and strategies that support the initiation and implementation of intersectoral action.

We considered all study designs and interventions that reflected different approaches to reducing health inequities, with universal interventions addressing the entire population (a horizontal approach) [7,13,16], targeted interventions selectively providing interventions to disadvantaged groups (a vertical approach) [7,13,16], and mixed approaches (“targeting with universalism”) directing extra benefits to disadvantaged groups within the context of a universal policy design [17].

## Methods

We used a rapid review method to synthesize the evidence of the impact of intersectoral action on the social determinants of health and health equity. Rapid reviews use streamlined traditional systematic review methods to help synthesize and communicate evidence within a shortened time frame [18].

### Search

Six electronic databases were searched in January 2012 for literature published between 2001 and 2011: Embase, MEDLINE, CINAHL, Social Sciences Abstracts, and the Cochrane and Campbell Libraries using search terms related to “intersectoral action”, “intersectoral collaboration”, “multisectoral collaboration” and public health. The detailed search strategy and grey literature search is included as an electronic Additional file 1. Additionally, content experts on the project advisory group and beyond (n = 6) were asked to identify studies likely to meet the inclusion criteria.

### Study selection

Two reviewers independently screened the titles and abstracts of all articles identified in the search. Articles with any differences in inclusion were passed into full-text screening. Two reviewers independently assessed full text papers, conflicts in inclusion were resolved by discussion and a third reviewer was involved if agreement was not reached. The inclusion criteria are presented in Table 1.

**Table 1:** Inclusion criteria

**Data type**	**Primary research, quantitative or qualitative data**
Participants	General population
Setting	Norway, Finland, Denmark, Sweden, Australia, New Zealand (NZ), Canada, the United States (US), or the United Kingdom (UK).
Publication date	January 2001 and January 2012
Health condition	Any
Intervention	Any intersectoral intervention involving public health
Comparator	Any
Outcomes	Any health outcome any measure of mortality and morbidity, healthcare utilization, adherence to healthcare, or quality of life.
	Any social determinant of health outcome
	Policy outcomes include societal-level legislative changes (e.g., laws, bills), as well as organizational-level policies, programs, and strategies to improve the social determinants of health and health equity.
Study type	Any
Dissemination type	Published journal paper and grey literature reports
Publication language	English and French

Any theoretical paper or commentary, study measuring only process outcomes, or interventions focused on only primary health care was excluded.

### Quality assessment

Two reviewers independently assessed the quality of included studies (see Table 2). The systematic review was assessed using AMSTAR [19-21], quantitative studies using a tool developed by the Effective Public Health Practice Project [22-25] and qualitative studies according to criteria developed by Letts, Wilkins, Law, Stewart, Bosch, & Westmorland [26]. The reviewers met to analyze their ratings, discuss differences, define terms, and reach consensus for conflicting responses for all included studies.

**Table 2:** Quality assessment results


**Systematic review (Shea et al. 2007)**[21]							**Smith**[27]
Q1. Was an a priori design provided?							Yes
The research question and inclusion criteria should be established before the conduct of the review.							
Q2. Was there duplicate study selection and data extraction?							Yes
There should be at least two independent data extractors, and a consensus procedure for disagreements should be in place.							
Q3. Was a comprehensive literature search performed? At least two electronic sources should be searched. The report must include years and databases used (e.g., Central, EMBASE, and MEDLINE). Key words and/or MESH terms must be stated, and where feasible the search strategy should be provided. All searches should be supplemented by consulting current contents, reviews, textbooks, specialized registers, or experts in the particular field of study, and by reviewing the references in the studies found.							Yes
Q4. Was the status of publication (i.e., grey literature) used as an inclusion criterion? The authors should state that they searched for reports regardless of their publication type. The authors should state whether or not they excluded any reports (from the systematic review), based on their publication status, language, etc.							Yes
Q5. Was a list of studies (included and excluded) provided? A list of included and excluded studies should be provided.							Yes
Q6. Were the characteristics of the included studies provided? In an aggregated form such as a table, data from the original studies should be provided on the participants, interventions and outcomes. The ranges of characteristics in all the studies analyzed (e.g. age, race, sex, relevant socioeconomic data, disease status, duration, severity, or other diseases should be reported.							Yes
Q7. Was the scientific quality of the included studies assessed and documented? ‘A priori’ methods of assessment should be provided (e.g., for effectiveness studies if the author(s) chose to include only randomized, double-blind, placebo controlled studies, or allocation concealment as inclusion criteria); for other types of studies alternative items will be relevant.							Yes
**Quantitative studies (Thomas et al) **[22]							
**Author**	**Selection bias**	**Study design**	**Confounders**	**Blinding**	**Data collection methods**	**Withdrawals/Dropouts**	**Global rating**
Bruzzese [33]	Weak	Strong	Strong	Moderate	Strong	Moderate	**Moderate**
Wills [29]	Strong	Moderate	Moderate	Moderate	Strong	Not applicable	**Moderate**
Findley [32]	Strong	Moderate	Strong	Moderate	Weak	Strong	**Moderate**
Jackson [31]	Strong	Moderate	Strong	Weak	Strong	Strong	**Moderate**
Hollar [30]	Moderate	Strong	Strong	Weak	Strong	Strong	**Moderate**
Freeman [28]	Moderate	Strong	Strong	Strong	Strong	Strong	**Strong**
Melvin [38]	Moderate	Moderate	Weak	Weak	Moderate	Not applicable	**Weak**
Sherring [35]	Moderate	Moderate	Weak	Weak	Strong	Moderate	**Weak**
Cheadle [34]	Weak	Moderate	Weak	Weak	Weak	Not applicable	**Weak**
Pechter [36]	Weak	Weak	Weak	Weak	Weak	Not applicable	**Weak**
Macnab [41]	Weak	Moderate	Strong	Moderate	Strong	Weak	**Weak**
Fazel [37]	Moderate	Moderate	Strong	Weak	Strong	Weak	**Weak**
Bailie [40]	Strong	Moderate	Strong	Weak	Weak	Moderate	**Weak**
Peifer [39]	Weak	Moderate	Weak	Weak	Weak	Not applicable	**Weak**
**Qualitative studies (Letts et al. 2007)**[26]						**Collie-Akers**[42]	**Metzel**[43]
Study purpose: Was the purpose and/or research question stated clearly?						Yes	Yes
Literature: Was relevant background literature reviewed?						Yes	Yes
Study design	What was the design?					Case study	Qualitative description
	Was a theoretical perspective identified?					Yes	Yes
Method(s) used						Document review and interviews	Interviews
Sampling	Was the process of purposeful selection described?					No	Yes
	Was sampling done until redundancy?					Not addressed	Not addressed
Was informed consent obtained?						Not addressed	Yes
Data Collection							
Descriptive clarity	Clear and complete description of site					Yes	Yes
	Clear and complete description of participants					Yes	Yes
	Role of researcher and relationship with participants					Yes	No
	Identification of assumptions and biases of researcher					No	No
Procedure rigour	Procedural rigour was used in data collection strategies					Yes	Yes
Data Analyses							
Analytical rigour	Data analyses were inductive					Yes	Yes
	Findings were consistent with and reflective of data					Yes	Yes
Auditability	Decision trial developed					Yes	Yes
	Process of analyzing the data was described adequately					No	Yes
Theoretical Connections	Did a meaningful picture of the phenomenon under study emerge?					Yes	Yes
Overall rigour							
Was there evidence of the four components of trustworthiness?	Credibility					Yes	Yes
	Transferability					Yes	Yes
	Dependability					Yes	Yes
	Confirmability					No	Yes
Conclusions and Implications							
Conclusions were appropriate given the study findings						Yes	Yes
The findings contributed to theory development and future practice/research						Yes	Yes

### Data extraction and analysis

Data extraction was conducted by one reviewer and was checked by another for completeness and accuracy. Table 3 includes a description of the criteria used for data extraction. The data are reported in a narrative format that includes information on the study design, the intervention, and the outcomes. Statistically significant and non-significant outcomes that were relevant to the review question are reported. Given the heterogeneity of the included studies, a meta-analysis would not have been appropriate, because outcome measures were not measured consistently across the included studies, and most studies did not include statistical analyses that would lend them to meta-analysis.

**Table 3:** Data extraction criteria

**Item**	**Description**
Location	Country
Setting	Rural, urban, organizational, local, regional, national
Population	Description of population if specified
Population health approach to addressing health equity	Interventions may be defined by their approach to reducing health inequities, with universal interventions addressing the entire population [5,16,27], targeted interventions selectively providing interventions to disadvantaged groups [5,16,27], and mixed approaches (“targeting within universalism”) directing extra benefits to disadvantaged groups within the context of a universal policy design [28].
Level of intervention	Interventions to advance health equity may be categorized by their approach to addressing the “upstream,” “midstream,” or “downstream” determinants of health [16,29,30].
	Interventions are classified as upstream interventions if they include reform of fundamental social and economic structures and involve mechanisms for the redistribution of wealth, power, opportunities, and decision-making capacities. Upstream interventions typically involve structural and system-level changes.
	Midstream interventions seek to reduce risky behaviours or exposures to hazards by influencing health behaviours or psychosocial factors and/or by improving material working and living conditions. Midstream interventions generally occur at the community or organizational level.
	Downstream interventions occur at the micro and/or individual level and mitigate the inequitable impacts of upstream and midstream determinants through efforts to increase equitable access to health care services.
Sectors	Description of sectors involved
Relationship between sectors	Based on four patterns of relationships in intersectoral action: information-sharing, cooperation, coordination, and integration [5]. An informative relationship is based on information sharing and exchange between sectors; cooperation refers to the achievement of greater efficiency through optimization of resources for the enforcement or implementation of policies or programs; coordination involves joint work among sectors for greater efficiency and effectiveness, generally the creation and integration of synergistic relationships and shared financing; and integration refers to approaching a new policy or program in conjunction with multiple sectors and requires the synthesis of objectives, administrative processes, resources, responsibilities, and actions;
Role of public health	Four roles for public health action on the social determinants of health to advance health equity include [31,32]:
	◦ “Reporting/ assessing on the health of populations and describing health inequalities and inequities and effective strategies to address those inequalities and inequities.
	◦ Modifying and orienting interventions to reduce health inequities including the unique needs and capacities of priority populations.
	◦ Engaging in community and multi-sectoral collaboration to address the health needs of priority populations through services and programs.
	◦ Leading/participating and supporting other stakeholders in policy analysis, development and advocacy for improvements in the health determinants/inequities”
Tools, strategies, and mechanisms	Tools may be described as catalysts that facilitate intersectoral action; mechanisms as institutional structures and arrangements; and strategies as a broader combination of planned actions or initiatives [8]
Social determinant of health	Description of social determinant of health addressed in intervention

## Results

### Search results

**Figure 1:**
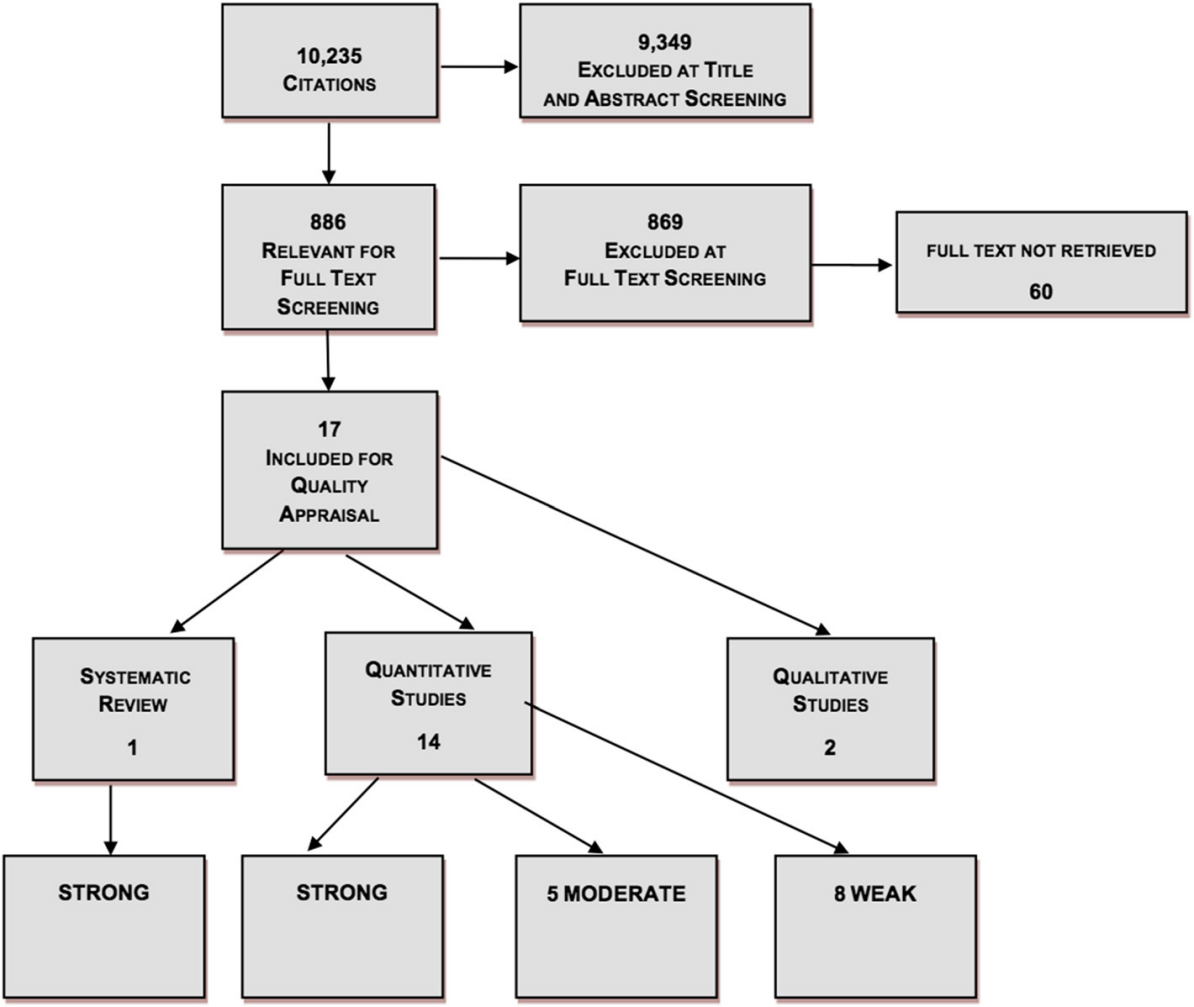
Search results.

The searches located 10,235 articles, including primary studies and systematic reviews (Figure 1). These went through title and abstract screening and 886 articles were deemed potentially relevant and underwent full-text screening for relevance testing. For 60 articles (0.6% of the total identified), we were unable to retrieve the full text; these articles were excluded at the full-text screening stage. 17 articles met the inclusion criteria: 1 systematic review, 14 quantitative studies, and 2 qualitative studies.

### Quality of included studies

We identified one strong systematic review [27]. Of the 14 primary studies, one was methodologically strong [28], five were of moderate quality [29-34], and eight were weak [35-41]. The quality assessment of two qualitative studies [42,43] and all other included studies are summarized in Table 2.

### Interventions

The characteristics of included studies are briefly described in Additional file 2: Table S1.

### Populations

All interventions involved individuals and communities that were experiencing social and/or economic disadvantages: children [28-30,32,33,37-41], socio-economically disadvantaged populations [28-30,32-34,36,38,39,42], racialized communities [30,32-34,42], refugee and/or immigrant populations [28-30], Aboriginal communities [40,41], and people with disabilities [35,43].

### Intervention settings

The majority of the interventions were implemented at the local community level, and in school, or workplace settings. Six interventions occurred within school settings [28,30,33,37,38,41]. One intervention was implemented within a workplace [36]. Seven studies focused on community-based interventions [27,29,32,34,39,40,42]. Three of these community-based interventions occurred within urban settings [32,34,42], and two occurred in remote and/or rural settings [29,40] and one [39] did not specify if it was a rural or urban setting. Three interventions occurred at the regional, district or state level [31,35,43].

### Population health approach to reducing health inequities

None of the studies included in this review evaluated a strictly universal intervention. Two interventions took a mixed approach by offering universal programming to all involved in the intervention and additional programming for specific groups [30,38]. Other studies investigated targeted interventions [27-29,31-33,35-37,39-43]. One study investigated a multi-component intervention that offered both universal and targeted programs and policies [34].

### Outcomes

A systematic review by Smith and colleagues [27], found that the design of partnership interventions and of the studies evaluating them meant it was difficult to assess the extent to which identifiable successes and failures were attributable to the partnerships. Included studies were of mixed methodological quality, typically short-term, and the majority were not designed specifically to assess the impact of partnerships on public health outcomes, including health equity. Their findings indicated that the impacts of intersectoral action on health equity are mixed and limited.

In this section, the findings of the primary studies are presented on the basis of how they intervened on the social determinants of health (i.e., upstream, midstream, or downstream interventions-see Table 3 for definition) [7,44,45].

### Upstream interventions

Two studies examined upstream interventions, one focusing on improving housing conditions [40] and the other on employment [43].

#### Housing

An evaluation of an Australian indigenous housing program assessed the impact of a building program on housing conditions for young children [40]. The study measured overcrowding (number of people per bedroom sleeping in the house), housing infrastructure (Failed Healthy Living Practice Score and Surveyor Function Score), and hygiene (Surveyor Condition Score). A moderate impact on improved housing infrastructure (5.6, CI [5.3, 6.0] to 4.4, CI [4.1, 4.8] (*p* = <.0001)), and no effect on overcrowding (3.4, (CI) [3.1, 3.6] to 3.2, CI [2.9, 3.4] (*p* = .102)) and hygienic conditions (4.1, CI [3.9, 4.4]; *p* = .605 at baseline and follow-up) were observed.

#### Employment

Metzel et al. [43] qualitatively examined the development and implementation of six interagency agreements between vocational rehabilitation and mental health organizations to support employment for people living with disabilities in six states in the US. Five of the six states reported an increase in supported employment for people with disabilities. Estimates from the various programs indicated a 25% yearly increase in employment from 1994 to 1999. More specifically, in 1997 there was an increase of 30%, with 200–300 young people benefiting from vocational assessment and employment opportunities, and between 1995 and 1996 there was an increase of 14%. Representatives from three of the states described an increase in coordination and cooperation (e.g., alteration of processes, systems change, and coordination of budgets).

### Midstream interventions

Eight studies reported on midstream interventions that addressed a range of social determinants of health: employment and working conditions [35,36], early childhood development [39], housing [31], physical and social environments [28,30,34,42], and food security [28].

#### Employment and working conditions

Two studies addressed employment and working conditions [35,36].

Sherring et al. [35] conducted a longitudinal cohort study to assess the impact of a supported employment intervention providing competitive employment for people with mental illness in Australia (*n* = 43). Overall, 76.7% (*n* = 33) of the participants obtained competitive employment at some point during the study, and after 24 months, 46.5% (*n* = 20) were still employed. The mean duration of employment was 24.7 weeks (standard deviation [*SD*] = 27.1, range 2.5–99.6), participants averaged 24.7 hours of work per week (*SD* = 12.8, range 3–40), and they earned AU$17.5/hour (*SD* = 4.9, range 7.6–30.4). Minimum wage was AU$13.74/hour at the time. Sherring et al. reported that employment outcomes were not significantly related to gender, age, or level of education (data not provided in the study) [33].

Pechter et al. [36] described how the Massachusetts Coalition for Occupational Safety and Health worked with a union of predominantly low-income, Spanish-speaking immigrant workers (*n* = 49; 35% of potential respondents), to assess workplace symptoms, hazards and equipment and to improve working conditions by reducing exposure to hazards. Five priority changes to the workplace environment were made.

#### Early childhood development: literacy

Peifer and Perez [39] sought to identify the impact of four coordinated, community-based early childhood literacy initiatives on parental behaviour among primarily low-income women in the US. Two samples were compared: 2001 (*n* = 300) and 2003 (*n* = 216). The comparison between the two time periods showed an increase in all early literacy behaviours (p values not provided). There was a 77% increase in the ratio of parents reporting that they showed books to their infants on a daily basis (53.67% in 2001, 69.44% in 2003). There was a 61.44% increase in the ratio of parents reading books aloud to their children on a daily basis (33% in 2001, 53.70% in 2003). The percentage of mothers who reported engaging in the Raising a Reader program was 4.3% in 2001 and 16.7% in 2003.

#### Housing

The Healthy Housing Programme, aimed to improve housing conditions in NZ [31]. Using an interrupted time series design, the study involved 9,736 residents in 3,410 households with a median of 2.3 years post-intervention data. Post-intervention hospital admissions for children up to 4 years old declined by 11% (hazard ratio [HR] = 0.89, CI [0.79, 0.99]); admissions among those 5–34 years old declined by 23% (HR = 0.77, CI [0.70, 0.85]); and there was no observed change in admissions among adults aged 35 years or older (HR = 1.04, CI [0.95, 1.15]). After the intervention, housing-related avoidable hospital admissions were 12% less for children up to 4 years old (HR = 0.88, CI [0.74, 1.05]), were reduced by 27% for those 5–34 years old (HR = 0.73, CI [0.58, 0.91]), and increased by 31% for those 35 years of age or older (HR = 1.31, CI [1.09, 1.56]).

#### Social and physical environments

Cheadle et al. [34] evaluated Steps to Health King County, a multi-project initiative conducted in an area of King County in Washington State in the US with a population of 352,836, of whom 14.4% were African American, 8.9% Hispanic or Latino, and 3.9% Vietnamese. More than 30% of residents lived below 200% of the Federal Poverty Line. The study reported outcomes from eight projects, which consisted of both midstream and downstream interventions (the downstream interventions are described in the next section). Projects received funding for midstream interventions for service integration and systems and policy change at the organizational, legislative, and regulatory levels. Although a few organizations engaged in policy and integration at the program level, most did not (numbers not specified). Program key informants noted that staff members were too busy managing day-to-day operations and that policy issues seemed too remote from their core mission of serving clients. Cross-program integration was described as modest and unsustained. Twenty-five organizational changes in schools and the community were attributed in full or in part to the efforts of the collaborative. The collaborative also engaged in 20 advocacy campaigns on local, state, and national issues, with mixed success.

Freeman et al. [28] assessed the effectiveness of a school-based break-time snacking initiative on the oral health of children attending schools in areas with low socio-economic status (SES) in Northern Ireland. This intervention was intended to change health behaviours and improve health outcomes by altering the school environment. At the end of the study, the intervention group (low SES) had a mean DMFT (decayed, missing, filled teeth index) score of 1.58, CI [1.28, 1.89]), whereas the control group (high SES) had a mean DMFT score of 0.065, CI [0.38, 0.93]. In addition, the DMFT in the intervention group (*n* = 99) changed from 1.13, CI [0.85, 1.40] in year 1 to 1.58, CI [1.28, 1.89] in year 2. There was also an increase in the number of filled permanent teeth among students from lower SES schools over time: mean 0.49, CI [0.20, 0.77] in year 1 and 1.05, CI [0.69, 1.14] in year 2.

Collie-Akers and colleagues [42] evaluated the impact of the Kansas City - Chronic Disease Coalition in the US, the goal of which was to reduce the risk of cardiovascular diseases and diabetes among African Americans and Hispanics. The study used a case study design to document changes in the community attributable to the work of the coalition. Of 729 events or activities facilitated by the Coalition, 321 instances of community change (new programs, policies, or practices) were reported. Of these, 75% were designed to reduce residents’ risk of both cardiovascular disease and diabetes, 13% to reduce the risk of diabetes, 6% to reduce the risk of cardiovascular disease, and 5% to address health care access or disparities. Providing information and enhancing skills constituted the most frequent strategy used (by 38% of the activities), followed by modifying access, barriers, and opportunities (27%); changing the consequences (14%); enhancing services and support (10%); and modifying policy (9%). Although no health outcomes were reported, given the early nature of the coalition’s activities at the time of publication, the authors noted that tracking community changes over time will help to link these changes to population health changes over the long term.

#### Social and physical environments and food security

Hollar et al. [30] conducted a controlled clinical trial of an elementary school–based obesity prevention program in Florida. The study involved a sample of 3,769 students (50.2% Hispanic, 33.4% white, 8.0% Black, and 8.4% other), 3,032 students in four intervention schools and 737 in one control school, with an average age of 8 years (range 4 to 13). In year 2, mean body mass index (BMI) declined by 1.73 (SD = 13.6) in the intervention schools and by 0.47 (SD = 12.1) in the control school (p = .007). Girls in the control group had an increase in mean systolic blood pressure, from 98.37 to 101.44 mm Hg (p < .001), and boys in both groups had an increase in systolic blood pressure (100.83 to 101.94 mm Hg in the intervention group and 99.28 to 101.93 mm Hg in the control group) (p < .0001). Diastolic blood pressure increased in both boys and girls in the intervention and control groups (p < .0001). A sub-sample of low-income students (n = 1,197; 68% Hispanic, 15% white, 9% Black, and 8% other) received free or reduced-cost school lunches. In this sub-sample, children in the intervention schools were more likely to reduce their BMI (p = .0013) and their weight (p < .011) than children in the control school over the 2-year intervention period. Math scores of students in the intervention group improved (p < .0005), and Hispanic and white children in intervention schools were more likely to have higher math scores (p < .001) than their counterparts in the control school. There was no observed change in math scores among Black students. Children in the intervention schools had higher reading scores than those in the control school in both years of the intervention (p < .08).

### Downstream interventions

All seven downstream interventions focused on access to health services or care [29,32-34,37,38,40].

#### Case coordination

The downstream interventions evaluated by Cheadle et al. [34] consisted of case coordination and case management, multi-session physical activity programs and health education for youth, training and education sessions for child care providers and community members, and bicycle safety promotion. Of case-managed patients, 45% established care with a primary care provider; in addition, there were 40% fewer emergency department visits among patients in the case management program after they were connected to a primary care provider, compared to the average for three comparison groups (0.79 vs.1.31 visits/year, p < .05), and the proportion of patients with poor diabetic control (hemoglobin A1c > 9) decreased from 78% before entering case management to 48% after (p < .05).

#### School readiness

A school readiness program, Before School Check, aimed to identify and address health, behavioural, social, or developmental concerns that might impact school performance and readiness in Hawke’s Bay, a largely rural community on the east coast of NZ. Wills et al. [29] measured the rate of referrals following training of pediatricians, nurses, public health staff, and academics to conduct the Before School Check and referrals for 4-year-old children. A range of tools were used to assess school readiness and to refer children to services as required. A total of 1,848 checks (84% of the cohort) were completed over a 10-month period, and the program maintained a 50% referral rate. Screening rates by income quintiles 1 to 5 (high to low) were Q1, 110%; Q 2 and Q3, 90% each; Q4, 80%, and Q5, 75% (no statistical analysis provided). The authors noted difficulties in recruiting children from low-income families, compared to children from higher SES families.

#### Mental health

One study described the establishment of a school-based mental health service for refugee children in the UK. [37] Using a pre/post survey design, Fazel et al. [37] assessed the impact of the service on students’ mental health using a 25-item Strengths and Difficulties Questionnaire (SDQ). The intervention group (*n* = 47) was made up of refugee students. Students in each of the control groups (ethnic, *n* = 47; white, *n* = 47) received no intervention. There were overall differences between the three groups (with refugee children scoring higher, but no significant difference between the two control groups) in SDQ total score (F [2, 138] = 6.6, p = .002) and in the scales for emotional symptoms (F [2, 138] = 11.5, p < .001) and peer problems (F [2, 138] = 4.2, p = .017). Over the study period (pre- vs. post-treatment), the total SDQ score in all groups decreased (F [1, 138] = 5.9, p = .016), with the greatest changes evident in the peer problems scale (F [1, 138] = 8.1, p = .005) and the hyperactivity scale (F [1, 138] = 3.9, p = .05). Hyperactivity scores decreased more in the refugee group than in the control groups (mean change –0.96 [SD = 2.40] vs. –0.10 [SD = 1.98]; *t* = 2.12, p = .037), with a suggestion of an effect in the emotional symptoms score (mean change –0.72 [SD = 2.63] vs. 0.03 [SD = 2.02]; *t* = 1.73, p = .088). At the end of the 1-year study period, refugee children continued to have significantly higher SDQ total scores (F [2, 138] = 4.7, p = .011), emotional symptom scores (F [2, 138] = 8.6, p < .001), and peer problem scores (F [2, 138] = 6.3, p = .002) than those in the control groups [35].

#### Oral health

Two studies focused on the provision of dental or oral health services [38]. A study of a school-based oral health program examined the impact of providing dental services to refugee students in the US [36]. In year 1, the program served 1,144 students and in year 2 it served 353 children. The percentage of children receiving preventive care increased from 52% in year 1 to 60% in year 2. In year 2, 212 children (60%) received preventive care, and 39 children (11%) received restorative care. The number of children receiving restorative care decreased by 11% in year 2 (no p values provided) [38].

Macnab et al. [41] conducted a cross-sectional study of a school-based dental health program in a rural, remote Aboriginal community in Canada (population 300). All children attending the community school (n = 26 at baseline and n = 40 at follow-up) participated in an oral health program. At the start of the program, the mean DMFT score was 5.5 (SD = 6.2) and at 3-year follow-up the mean score was 6.1 (SD = 8.5) (p < .05). Children assessed both before and after the intervention, (n = 13) had improvements in dmfs/DMFS (total number of decayed, missing due to caries, and filled surfaces: primary/permanent) (p < .005) and dmft/DMFT (p < .05) scores.

#### Immunization

Findley et al. [32] assessed the impact of Start Right, a community-based immunization promotion program of outreach and tracking for children under 5 years of age in Northern Manhattan, in New York City in the US. Intervention participants were children 19 to 35 months of age as of April 16, 2004 (n = 1,502), and rates were compared with the National Immunization Survey of 2003. Over a 2-year period, immunization rates improved, and there was no significant difference in immunization rates between Start Right participants (80.5%) and the national population (79.4%) (t = 0.87). The immunization rate among African Americans in the study (n = 281) was 78.4% (SD = 4.7), compared to the US immunization rate for African Americans of 73.3% (SD = 3.3) (t = 2.90). Among Latino participants (n = 1,122), the immunization rate was 83.7% (SD = 4.9), compared to the national rate (77.0% [SD = 2.1], t = 2.32) and the local rate (73.7% [SD = 9.5%], t = 3.75) for Latino populations. Latino children were more likely to be up-to date than were African American children (OR = 9.81, [CI = 1.1, 2.1]). The overall immunization rate among Start Right participants increased from 46% in 2003 to 80.5% in 2004.

#### Asthma management

A controlled clinical trial measured the impact of a school-based asthma intervention for low- income ethnic minority families in New York City [33]. At 2 years post-intervention, control students had had fewer admissions to hospital in the previous 12 months (control 0.1 [SD = 0.3] vs. intervention 0.2 [SD = 0.6], p < .05) [31].

### Relationships and roles

The interventions described involved a number of different sectors, roles, and relationships. Although we attempted to categorize the nature of the relationships between sectors involved in an intervention, these relationships were not always clearly defined. Where provided these are reported in Additional file 2: Table S1.

### Tools, mechanisms, and strategies

The initiation and implementation of the intersectoral interventions were supported by a number of tools, mechanisms, and strategies, but these supporting elements were not always described in the included studies. Where provided these are reported in Additional file 2: Table S1.

## Discussion

Intersectoral action for the SDH is a key approach to improve health equity [46]. Upstream or structural interventions are likely to have the greatest impact in terms of reducing health inequities because they change the underlying conditions in which people live, work, and play [47,48]. Only two out of the 16 included primary studies addressed upstream determinants of health, eight addressed midstream determinants, and seven addressed downstream determinants.

The strongest effects were observed with more downstream interventions for population health outcomes such as intersectoral collaborations to improve immunization rates and oral health among vulnerable populations. Midstream intersectoral interventions have shown moderate to no impact on the SDH and health equity. The association between upstream interventions and health outcomes is less conclusive. This is likely because the impact of upstream interventions on health equity and SDH is more difficult to evaluate.

While all of the included studies focused on populations experiencing social and/or economic disadvantage few of these studies specifically described assessing and comparing the impacts of interventions in marginalized groups with the impacts of such interventions in other groups within the population. The majority of studies did not specifically analyze the health equity implications of the interventions in terms of multiple factors of disadvantage. It is possible that some initiatives would improve the health of marginalized populations without changing the gap between marginalized and privileged groups. Furthermore, there was an emphasis on midstream and downstream interventions compared to upstream and structural intervention. For example, none of the included studies that focused on racialized communities addressed the issue of institutionalized racism. Previous work has noted the challenge of addressing upstream determinants of health [49].

To understand the impact of intersectoral initiatives on various populations, the equity analysis in interventions should be strengthened [49]. Such analysis includes incorporating approaches that assess the change in health for the targeted group and reference to how any observed improvement affects the divide between the marginalized group and more privileged groups. One approach to narrowing the health divide considers the gap between those who are disadvantaged and those who are advantaged and strives to reduce the difference in health status between these extremes of the social scale. Additionally, interventions can focus on reducing social inequities throughout the whole population and creating better opportunities for health across the socio-economic continuum [9].

The majority of included studies evaluated setting-specific (e.g., schools and workplaces), local, and district-level interventions. Few studies examined regional-level interventions, and none explored large-scale policy interventions. As such, findings may not be generalizable to other populations or settings.

Given that the relationships between sectors and how these relationships contributed to outcomes was not clearly articulated in the description of interventions, it is difficult to attribute the effectiveness of initiatives or lack thereof to intersectoral action. Successes and failures of the programs and policies may have been the result not of partnership, but of other contextual factors. The included studies generally provided few details about the process, context, successes, and challenges of the intersectoral interventions and how these were related to the observed outcomes. For most interventions, it is unclear whether the same outcome would have been observed if only one sector had been responsible for development and implementation.

Context-specific, complex, and process-oriented approaches such as intersectoral action require similarly appropriate mechanisms for assessing impact [6,50]. The complexity of evaluating the impact of intersectoral action on the SDH to improve health equity calls for more rigorous approaches to evaluate intersectoral action along a continuum, taking into account intersectoral processes, and the implementation and health equity impacts of interventions. Long-term, large, controlled quantitative studies, as well as mixed-methods studies (which would take into account contextual factors) and well-designed qualitative studies involving the intended beneficiaries, are required to better understand the impact of intersectoral action on health equity.

### Limitations of available evidence

This expedited review had some limitations related to the primary studies and the review methodology. The methodological quality of the included primary studies limits the ability to draw concrete conclusions. In particular, many of the primary studies had the potential for selection bias. Blinding was not often used in the studies, which may reflect the type of interventions being investigated. Furthermore, the majority of interventions were short-term and may not have had sufficient time for impacts to be observed. The sectors involved in an intervention were not always explicitly described in the published studies considered for inclusion.

The shortened time frame for the review (less than 3 months) meant that the time available to retrieve articles was reduced. Further, the limited time period prevented hand-searching relevant journals. However, we adhered to most of the criteria for conducting a full systematic review.

## Conclusions

The purpose of this expedited review was to examine the state of the published evidence regarding the impact of intersectoral action as a public health practice on health equity through action on the SDH. The body of literature on intersectoral action as a promising practice is mixed, revealing moderate to no effect on the SDH. The evidence on the impact of intersectoral action on health equity is even more limited. We found that much of the available literature is descriptive and that programs are not rigorously evaluated. Furthermore, there is a major gap in the literature, with mechanisms linking intersectoral processes to observed outcomes being mostly absent. The majority of outcome evaluations described within this review were not methodologically strong, a limitation that should temper any conclusions drawn from the review.

### For practice and policy

•Collaborations between public health and other sectors show promise in creating supportive environments, as well as in enhancing access to services for marginalized populations. However, on their own, intersectoral initiatives that focus on downstream determinants are unlikely to eliminate disparities. There is a need for more multi-level interventions that address structural determinants of health across the whole population.

•Intersectoral initiatives need to include a comprehensive equity analysis to identify any populations that are positively or negatively affected and the contexts under which such effects occur. This is important to ensure that interventions do not increase population health inequities.

•Publishing findings from program and policy interventions contributes to the evidence base about intersectoral action for health equity. Adequate funding and partnerships with researchers support organizational capacity to collect data for rigorous evaluation.

•Funding for initiatives was reported as an important mechanism supporting the initiation, implementation, and evaluation of initiatives.

### For research

•Methodological issues such as selection bias, blinding, and sample size should be addressed in future studies on intersectoral action.

•Rigorous evaluation of intersectoral action is needed, particularly for upstream interventions. Evaluations of the health equity impacts of intersectoral action should include prospective and, where possible, controlled designs with sufficiently long follow-up to identify trends. Evaluations of program and policy interventions must include both empirical outcome measures and descriptions of intersectoral activities, roles, and responsibilities. Creating an interdisciplinary body of knowledge about how to evaluate intersectoral action, along with supporting tools, will help strengthen the evidence base for intersectoral action on health equity and the social determinants of health.

•Papers reporting on the outcome of intersectoral interventions need to include more detail about the nature of intersectoral activities such as the nature of the interventions, the roles and responsibilities of various sectors and the impact this may have on the observed outcomes.

•Academic and practitioner partnerships are beneficial for evaluating intervention.

•Further research on the cost-effectiveness of intersectoral action is required

## Competing interest

The authors declare that they have no competing interest.

## Authors’ contributions

SNE and HM contributed to the design, appraisal, analysis and writing of the review. Both authors read and approved the final manuscript.

## Pre-publication history

The pre-publication history for this paper can be accessed here:

http://www.biomedcentral.com/1471-2458/13/1056/prepub

## Supplementary Material

Additional file 1 **Search Strategy.** Comprehensive search strategy. Click here for file

Additional file 2: Table S1 Description of included studies. Click here for file
