# Supplementary material for: Intersectoral action for health equity: a rapid systematic review
Source: BMC Public Health. 2013 Nov 9;13:1056. doi: 10.1186/1471-2458-13-1056 (PMC3830502; doi:10.1186/1471-2458-13-1056)
Supplement: Additional file 1 — Search Strategy. Comprehensive search strategy. [file 1471-2458-13-1056-S1.doc]

## Comprehensive Search Strategy

**Medline-OVID**

January 5, 2012

1. (multisectoral or multi-sectoral).tw.

2. (collaboration or collaborative).tw.

3. (cooperation or co-operation or cooperative or co-coperative).tw.

4. cross-sectoral.tw.

5. (horizontal adj3 management).tw.

6. (horizontal management or horizontal organization).tw.

7. whole of government.tw.

8. joined up.tw.

9. (interministerial or inter-ministerial).tw.

10. (interagency or inter-agency).tw.

11. (integrated adj (health or healthcare)).tw.

12. (intersector* or inter-sector*).tw.

13. (interdepartmental or inter-departmental).tw.

14. or/1-13

15. *interprofessional relations/ or *interdisciplinary communication/

16. *interdepartmental relations/ or *interinstitutional relations/

17. *Cooperative Behavior/

18. *"Delivery of Health Care, Integrated"/og, td [Organization & Administration, Trends]

19. multi-agency.tw.

20. or/15-19

21. 14 or 20

22. public health.mp.

23. public health/ or preventive medicine/

24. exp *"delivery of health care"/og, td

25. Community Medicine/og [Organization & Administration]

26. exp *Community Health Services/og, td [Organization & Administration, Trends]

27. public health administration/ or *health services accessibility/

28. or/22-27

29. Healthcare Disparities/

30. ((health or healthcare) adj2 (equity or inequit* or equality or inequalit* or disparit*)).tw.

31. "social determinants".tw.

32. exp *Socioeconomic Factors/

33. "socioeconomic determinants".tw.

34. socioeconomic factors.tw.

35. or/29-34

36. 28 or 35

37. 21 and 36

38. "health in all policies".tw.

39. 37 or 38

40. animals/

41. 39 not 40

42. limit 41 to (english or french)

43. limit 42 to (comment or editorial or in vitro or letter or video-audio media or webcasts)

44. 42 not 43

45. Developing Countries/

46. exp africa/ or exp caribbean region/ or exp central america/ or exp latin america/ or south america/ or exp antarctic regions/ or exp asia/

47. or/45-46

48. 44 not 47

49. limit 48 to yr="2001 -Current"

50. limit 49 to "review articles"

51. meta-analysis.pt,ti,ab,sh.

52. (meta anal$ or metaanal$).ti,ab,sh.

53. ((methodol$ or systematic$ or quantitativ$) adj3 (review$ or overview$ or survey$)).ti.

54. ((methodol$ or systematic$ or quantitativ$) adj3 (review$ or overview$ or survey$)).ab.

55. ((pool$ or combined or combining) adj (data or trials or studies or results)).ti,ab.

56. (medline or embase or cochrane or pubmed or pub med).ti,ab.

57. or/54-56

58. review.pt,sh.

59. 57 and 58

60. or/51-53

61. 59 or 60

62. 49 and 61

63. 50 not 62

64. 49 not 63

**Embase-OVID**

January 6, 2012

1. (multisectoral or multi-sectoral).tw.

2. (collaboration or collaborative).tw.

3. (cooperation or co-operation or cooperative or co-coperative).tw.

4. cross-sectoral.tw.

5. (horizontal adj3 management).tw.

6. (horizontal management or horizontal organization).tw.

7. whole of government.tw.

8. joined up.tw.

9. (interministerial or inter-ministerial).tw.

10. (interagency or inter-agency).tw.

11. (integrated adj (health or healthcare)).tw.

12. (intersector* or inter-sector*).tw.

13. (interdepartmental or inter-departmental).tw.

14. or/1-13

15. *public relations/

16. *interdisciplinary communication/

17. exp *cooperation/

18. integrated health care system/

19. multi-agency.tw.

20. or/15-19

21. 14 or 20

22. community medicine/ or preventive medicine/ or public health/

23. public health.mp.

24. health care disparity/

25. ((health or healthcare) adj2 (equity or inequit* or equality or inequalit* or disparit*)).tw.

26. or/22-25

27. 21 and 26

28. "social determinants".tw.

29. "socioeconomic determinants".tw.

30. *"social aspects and related phenomena"/ or *social aspect/ or exp *social status/ or exp *socioeconomics/

31. or/28-30

32. 21 and 31

33. "health in all policies".tw.

34. 27 or 32 or 33

35. animal/ or animal experiment/

36. 34 not 35

37. limit 36 to (english or french)

38. limit 37 to (book or book series or conference abstract or editorial or letter or note)

39. 37 not 38

40. limit 39 to yr="2001 -Current"

41. limit 40 to (meta analysis or "systematic review")

42. limit 40 to "review"

43. meta analysis/

44. meta-analysis.ti,ab.

45. (meta anal$ or metaanal$).ti,ab.

46. ((methodol$ or systematic$ or quantitativ$) adj3 (review$ or overview$ or survey$)).ti.

47. ((methodol$ or systematic$ or quantitativ$) adj3 (review$ or overview$ or survey$)).ab.

48. ((pool$ or combined or combining) adj (data or trials or studies or results)).ti,ab.

49. (medline or embase or cochrane or pubmed or pub med).ti,ab.

50. or/47-49

51. review.pt,sh.

52. 50 and 51

53. or/43-46

54. 52 or 53

55. 40 and 54

56. 41 or 55

57. 42 not 56

58. 40 not 57

**Cochrane Central-OVID**

January 31, 2012

1. (multisectoral or multi-sectoral).tw.

2. (collaboration or collaborative).tw.

3. (cooperation or co-operation or cooperative or co-coperative).tw.

4. cross-sectoral.tw.

5. (horizontal adj3 management).tw.

6. (horizontal management or horizontal organization).tw.

7. whole of government.tw.

8. joined up.tw.

9. (interministerial or inter-ministerial).tw.

10. (interagency or inter-agency).tw.

11. (integrated adj (health or healthcare)).tw.

12. (intersector* or inter-sector*).tw.

13. (interdepartmental or inter-departmental).tw.

14. or/1-13

15. *interprofessional relations/ or *interdisciplinary communication/

16. *interdepartmental relations/ or *interinstitutional relations/

17. *Cooperative Behavior/

18. *"Delivery of Health Care, Integrated"/og, td [Organization & Administration, Trends]

19. multi-agency.tw.

20. or/15-19

21. 14 or 20

22. public health.mp.

23. public health/ or preventive medicine/

24. exp *"delivery of health care"/og, td

25. Community Medicine/og [Organization & Administration]

26. exp *Community Health Services/og, td [Organization & Administration, Trends]

27. public health administration/ or *health services accessibility/

28. or/22-27

29. Healthcare Disparities/

30. ((health or healthcare) adj2 (equity or inequit* or equality or inequalit* or disparit*)).tw.

31. "social determinants".tw.

32. exp *Socioeconomic Factors/

33. "socioeconomic determinants".tw.

34. socioeconomic factors.tw.

35. or/29-34

36. 28 or 35

37. 21 and 36

38. "health in all policies".tw.

39. 37 or 38

40. animals/

41. 39 not 40

42. limit 41 to (english or french)

43. limit 42 to (comment or editorial or in vitro or letter or video-audio media or webcasts)

44. 42 not 43

45. Developing Countries/

46. exp africa/ or exp caribbean region/ or exp central america/ or exp latin america/ or south america/ or exp antarctic regions/ or exp asia/

47. or/45-46

48. 44 not 47

49. limit 48 to yr="2001 -Current"

**CINAHL-EBSCO**

January 7, 2012

S1 TX ( multisectoral OR multi-sectoral ) OR TX ( collaboration OR collaborative ) OR TX ( cooperation OR co-operation OR cooperative OR co-coperative ) OR TX cross-sectoral

S2 TX horizontal N3 management OR TX ( horizontal management OR horizontal organization ) OR TX whole of government OR TX joined up

S3 TX ( interministerial OR inter-ministerial ) OR TX ( interagency OR inter-agency ) OR TX ( intersector* OR inter-sector* ) OR TX ( interdepartmental OR inter-departmental )

S4 TX integrated health OR TX intergrated healthcare

S5 (MM "Interprofessional Relations")

S6 (MM "Interdepartmental Relations") OR (MM "Interinstitutional Relations")

S7 (MM "Cooperative Behavior")

S8 (MM "Health Care Delivery, Integrated")

S9 "multi-agency"

S10 S1 or S2 or S3 or S4

S11 S5 or S6 or S7 or S8 or S9

S12 S10 or S11

S13 (MM "Preventive Health Care") OR (MH "Public Health")

S14 (MH "Public Health Administration")

S15 (MM "Community-Institutional Relations") OR (MM "Community Networks/AM")

S16 (MM "Community Health Services+")

S17 TX public health

S18 S13 or S14 or S15 or S16 or S17

S19 "economic disparities"

S20 "healthcare disparities"

S21 MM "Health Services Accessibility"

S22 (MM "Socioeconomic Factors+")

S23 TX social determinants

S24 TX socioeconomic determinants

S25 TX health equity OR TX health inequ* OR TX health equality OR TX health disparit*

S26 TX healthcare equity OR TX healthcare inequ* OR TX healthcare equality OR TX healthcare disparit*

S27 TX health care equity OR TX health care inequ* OR TX health care equality OR TX health care disparit*

S28 S19 or S20 or S21 or S22 or S24 or S25 or S26 or S27

S29 S18 or S28

S30 S12 and S29

S31 S12 and S29 Limiters - Published Date from: 20010101-20120131; Human; Language: English, French

S32 S12 and S29 Limiters - Publication Type: Anecdote, Biography, Book, Book Chapter, Book Review, Editorial, Letter, Masters Thesis, Obituary, Pamphlet, Website

S33 S31 NOT S32

S34 MH developing countries

S35 S33 NOT S34

S36 TI africa OR TI India OR TI Asia

S37 S35 NOT S36

**Social Science Abstracts-EBSCO**

January 17, 2012

|  | **Query** | **Limiters/Expanders** |
| --- | --- | --- |
| S35 | S31 NOT S34 | Search modes - Boolean/Phrase |
| S34 | S32 or S33 | Search modes - Boolean/Phrase |
| S33 | TI India* OR TI Africa* OR TI China OR TI Asia* OR TI Latin America* OR TI chinese | Limiters - Publication Date: 20010101-20120131  Search modes - Boolean/Phrase |
| S32 | SU developing countries | Limiters - Publication Date: 20010101-20120131  Search modes - Boolean/Phrase |
| S31 | S28 or S29 | Limiters - Publication Date: 20010101-20120131  Search modes - Boolean/Phrase |
| S30 | S28 or S29 | Search modes - Boolean/Phrase |
| S29 | SU Integrated delivery of health care | Search modes - Boolean/Phrase |
| S28 | S8 and S14 | Search modes - Boolean/Phrase |
| S27 | S8 and S26 | Search modes - Boolean/Phrase |
| S26 | S24 and S25 | Search modes - Boolean/Phrase |
| S25 | TX health OR TX healthcare OR TX health care OR SU health care OR SU healthcare | Search modes - Boolean/Phrase |
| S24 | S15 or S16 or S17 or S18 or S19 or S20 or S21 or S22 or S23 | Search modes - Boolean/Phrase |
| S23 | TX healthcare equit* OR TX health care inequ* OR TX health care dispar* OR TX health care equal* OR TX health care inequal* | Search modes - Boolean/Phrase |
| S22 | TX healthcare equit* OR TX healthcare inequ* OR TX healthcare dispar* OR TX healthcare equal* OR TX healthcare inequal* | Search modes - Boolean/Phrase |
| S21 | TX health equit* OR TX health inequ* OR TX health dispar* OR TX health equal* OR TX health inequal* | Search modes - Boolean/Phrase |
| S20 | SU health indicators | Search modes - Boolean/Phrase |
| S19 | TX "socioeconomic determinants" OR TX "socioeconomic factors" | Search modes - Boolean/Phrase |
| S18 | SU socioeconomic determinants OR SU socioeconomic factors | Search modes - Boolean/Phrase |
| S17 | TX "social determinants" | Search modes - Boolean/Phrase |
| S16 | SU income distribution | Search modes - Boolean/Phrase |
| S15 | SU health and social status | Search modes - Boolean/Phrase |
| S14 | S9 or S10 or S11 or S12 or S13 | Search modes - Boolean/Phrase |
| S13 | SU community health services | Search modes - Boolean/Phrase |
| S12 | "preventive medicine" | Search modes - Boolean/Phrase |
| S11 | SU health services accessibility | Search modes - SmartText Searching |
| S10 | SU health promotion | Search modes - Boolean/Phrase |
| S9 | SU public health administration OR SU public health OR TX public health | Search modes - Boolean/Phrase |
| S8 | S1 or S2 or S3 or S4 or S5 or S6 or S7 | Search modes - Boolean/Phrase |
| S7 | SU Interprofessional cooperation OR SU health care teams | Search modes - Boolean/Phrase |
| S6 | SU Cooperative Behavior | Search modes - Boolean/Phrase |
| S5 | TX ( (interdepartmental or inter-departmental) ) OR TX integrated health OR TX integrated healthcare OR TX "integrated health care" | Search modes - Boolean/Phrase |
| S4 | ( (interministerial or inter-ministerial) ) OR ( (interagency or inter-agency) ) OR ( (intersector* or inter-sector*) ) | Search modes - Boolean/Phrase |
| S3 | TX horizontal N2 organization OR TX "whole of government" OR TX "joined up" | Search modes - Boolean/Phrase |
| S2 | horizontal N3 management | Search modes - Boolean/Phrase |
| S1 | TX ( (multisectoral or multi-sectoral) ) OR TX ( (collaboration or collaborative) ) OR TX ( (cooperation or co-operation or cooperative or co-operative) ) OR TX cross-sectoral | Search modes - Boolean/Phrase |

## Grey Literature Search

***Search Terms:*** *intersectoral, inter-sectoral, interagency, inter-agency, collaboration, “health in all”, “joined up”*

<http://cadth.ca/en/cadth> Canadian Agency for Drugs and Technologies in Health

<http://www.chspr.ubc.ca/node/106> UBC Centre for Health Services and Policy Research

[http://www.phac-aspc.gc.ca](http://www.phac-aspc.gc.ca/) Public Health Agency of Canada

[http://www.apha.ab.ca](http://www.apha.ab.ca/) Alberta Public Health Association

<http://www.gov.mb.ca/health/index.html> Manitoba Health

<http://www.phabc.org/> Public Health Association of BC

[http://www.actnowbc.ca](http://www.actnowbc.ca/) Act Now BC

[http://www.opha.on.ca](http://www.opha.on.ca/) Ontario Public Health Association

<http://www.aohc.org/> Association of Ontario Health Centres

<http://www.health.gov.nl.ca/health/> Newfoundland and Labrador, Department of Health and Community Services

<http://www.gov.ns.ca/DHW/> Nova Scotia, Department of Health and Wellness

<http://www.health.alberta.ca/> Government of Alberta, Health and Wellness

Saskatchewan [Health Quality Council](http://www.hqc.sk.ca/portal.jsp?gy7VlCeoNoTwE6QiGDF8ezBIzBf0QfLQkUwK4QBZaJvzXsUtFh9NC4zOVcA+lmY4)

<http://www.ices.on.ca/index.html> Institute for Clinical Evaluative Sciences

<http://www.ihe.ca/publications/library> Institute of Health Economics, Health Technology Assessment Unit, Alberta

<http://www.dh.gov.uk/health> UK, Department of Health

http://[www.institute.nhs.uk](http://www.institute.nhs.uk/) UK , National Health Service Institute for Innovation and Improvement

<http://kingsfund.koha-ptfs.eu/> UK, The King’s Fund Library Database

<http://www.health.govt.nz/> New Zealand, Ministry of Health

<http://www.pha.org.nz/> Public Health Association of New Zealand

<http://library.aifs.gov.au/> Australian Institute of Family Studies Library

<http://www.sweden.gov.se/sb/d/2061> Sweden, Ministry of Health and Social Affairs

<http://www.fhi.se/en/> Swedish National Institute of Public Health

<http://www.regjeringen.no/en/dep/hod.html> Norway, Ministry of Health and Care Services

<http://www.fhi.no/eway> Norwegian Institute of Public Health

<http://dsi.dk/english/> Danish Institute for Health Services Research

[http://www.si-folkesundhed.dk/Om%20instituttet.aspx](http://www.si-folkesundhed.dk/Om instituttet.aspx) Danish National Institute of Public Health

<http://www.ktl.fi/portal/english/ktl/> Finland, National Institute for Health and Welfare
