# Supplementary material for: Intersectoral action for health equity: a rapid systematic review
Source: BMC Public Health. 2013 Nov 9;13:1056. doi: 10.1186/1471-2458-13-1056 (PMC3830502; doi:10.1186/1471-2458-13-1056)
Supplement: Additional file 2: Table S1 — Description of included studies. [file 1471-2458-13-1056-S2.doc]

| **Additional file 2: Table S1: Included Studies** | | | | | | | | | | | | | | | |
| --- | --- | --- | --- | --- | --- | --- | --- | --- | --- | --- | --- | --- | --- | --- | --- |
| **Author** | **Title** | | **Methods** | **Location** | **Participants** | **Intersectoral action** | **Interventions** | **Outcomes** | **Subgroup analysis** | **Tools, mechanisms, and strategies for intersectoral action** | **Social determinant of health** | **Level of intervention** | **Population health approach to health equity** | | **Costs** |
| Bailie et al. 2011^40^ | Evaluation of an Australian indigenous housing programme: community level impact on crowding, infrastructure function and hygiene | | **Design:**  Cohort **Follow up:** On average 10 months after occupation of new houses  **Years of data collected**: 2 years | **Country:** Australia **Setting:** Community | **Sample:**  418 children living in 185 houses **Characteristics:** Households with children  **Race/ethnicity**: Indigenous Geographic setting: Rural or remote communities | **Time frame:** 2003 to 2007 **Sectors**: Public health, primary health care, academia, housing agencies,  Aboriginal councils Pattern of relationships between sectors: Integration **Activities and relationships:** Not specified  **Role of public health:** Not specified  **Public health individuals involved**: Not specified | Housing Improvement and Child Health Study: conducted in the 10 Northern Territory communities where there was the greatest construction of new houses by the Environmental Health Program of the Australian Government’s National Aboriginal Health Strategy and other large infrastructure programs over the period 2004–2005.  Additional housing was constructed to meet specific housing standards that were significantly more rigorous than standards applied in these communities over previous decades.  The average number of new houses to be constructed in each of the 10 communities was 11 (range 7–15). A small number of uninhabitable houses were earmarked for demolition. No concurrent renovation programs or hygiene promotion activities were conducted over the study period, so the housing intervention essentially consisted of the construction of a defined number of new houses.  The program’s impact was assessed using house infrastructure surveys and structured interviews with the main householder in all homes with young children in the 10 remote Australian indigenous communities. | At follow-up, there was a small (non-significant) decrease in the mean number of people per bedroom sleeping in the house on the night before the survey, from 3.4, confidence interval [CI] [3.1, 3.6] at baseline to 3.2, CI [2.9, 3.4] at follow-up (natural logarithm transformed t test, t = 13, p = .102). From baseline to follow-up, there was no significant change in the composition of households in terms of the numbers of younger and older children and adults. The Kruskal–Wallis test showed a significant difference in Failed Healthy Living Practice score (housing infrastructure) between baseline (mean 5.6, CI [5.3, 6.0]) and follow-up (mean 4.4, CI [4.1, 4.8]) (χ2 = 22.8, p < .001).  The Kruskal–Wallis test showed a marginally statistically significant difference in the Surveyor Function Score (housing infrastructure) between baseline (mean 3.8, CI [3.5, 4.0]) and follow-up (mean 3.4, CI [3.1, 3.6]) (χ2 = 3.9, p = .047). The Kruskal–Wallis test confirmed that there was no evidence of improvement in overall Surveyor Condition Score (hygienic conditions) (baseline mean 4.1, CI [3.9, 4.4]; follow-up mean 4.1, CI [3.9, 4.4]; χ2 = 0.3, p = .605). | None | **Initiation:** Policy: National Aboriginal Health Strategy **Implementation:** Housing standards | Housing | Upstream | Targeted | | Not specified |
| Bruzzese et al. 2006^33^ | | Using school staff to establish a preventive network of care to improve elementary school students’ control of asthma | **Design:** Controlled clinical trial Length of follow-up: 1 year, 2 years  **Length of follow-up:** 1 year, 2 years | **Country:** United States **Setting:** School | **Sample:** *n* = 591 students with prior asthma diagnosis (kindergarten to grade 5) and their caregivers (intervention: *n* = 307; control: *n* = 284) **Characteristics:**  Age: mean 7.8 years (SD = 1.4)  Income and race/ethnicity: eligibility criteria for schools specified that more than 50% of students had to be receiving free lunch and more than 67% had to be from ethnic minorities | **Time frame:** 1998–2001  **Sectors:** Public health, primary health care, education, academia  **Pattern of relationships between sectors:** Coordination  **Sectors:** Public health, primary health care, education, academia  **Pattern of relationships between sectors:** Coordination **Activities and relationships:** Creation of school health team, including full-time school nurse, school physician, and public health assistant; school teacher or administrator; and a parent. Academic partner provided training for school health team. Prevention activities were implemented by school health team with support from academic partner.  **Role of public health**: Training school staff and primary care providers.  **Public health individuals involved:** Public health nurse | The school health team participated in a 3-day workshop led by Columbia University staff during the summer. During the early fall, the school health team and Columbia University staff trained teachers in a single 45-minute session about asthma and their role in helping children manage asthma at school. Columbia University staff and New York City Department of Health and Mental Hygiene physicians also trained students’ primary care providers during the fall and early winter in state-of-the-art preventive therapy, communication, and patient education strategies, and procedures for establishing medication plans in schools using the Physician Asthma Care Education program. Nurses (a) called families to confirm case-detection information, further assess children’s asthma severity and health care needs, and provide caregivers with asthma education as needed; (b) sent sample treatment plans to primary care providers on the basis of students’ asthma severity, consistent with National Heart, Lung and Blood Institute guidelines, and blank treatment plans; (c) encouraged caregivers and primary care providers to complete required forms when medication was needed at school; and (d) referred families for medical care if needed. Additionally, nurses conveyed instructions from the management plans to teachers. | While case detection helped nurses to identify additional students with asthma and nurses increased the amount of time spent on asthma-related tasks, primary care providers did not change their medical management of asthma. Few improvements in health outcomes were achieved. Relative to controls, at 12 months after the intervention, students in the intervention group had a reduction in activity limitations due to asthma (–35% vs. –9%, *p* < .05) and more days without symptoms (26% vs. 39%, *p* = .06). The intervention had no impact on use of urgent health care services, school attendance, or caregiver’s quality of life, and there were no improvements at 24 months after the intervention. | None | **Initiation**: Not specified  **Implementation**: Creation of a school health team | Health services | Downstream | Targeted | Not specified | |
| Cheadle et al. 2011^34^ | | The impact of a community-based chronic disease prevention initiative: evaluation findings from Steps to Health King County | **Design:** Cohort **Follow up:**  Post intervention, 1 to 2 years **Years of data collected:** Not specified | **Country:** United States **Setting**: Community-based; mixed urban and suburban | **Sample:** *n* = 63,780 area residents (for all interventions)  **Characteristics:**  Race/ethnicity of area residents: 14.4% African American, 8.9% Hispanic or Latino, 3.9% Vietnamese  Income of area residents: More than 30% of residents below 200% of the Federal Poverty Line  Interventions focused on people with household income less than 200% of the federal poverty level who spoke English, Spanish, and/or Vietnamese | **Time frame:** 2003-2009 **Sectors:**  Public health, primary health care, academic, education, non-governmental organizations **Patterns of relationships between sectors:** Cooperation, coordination, integration **Activities and relationships:**  Leadership team: Public Health – Seattle & King County and 75 representatives of community-based organizations, hospitals, local government agencies, universities, school districts, etc.; implementation by all participating organizations, with financial and technical support from Public Health – Settle & King County; evaluation team: Centre for Community Health and Evaluation, Public Health – Seattle & King County staff, University of Washington **Role of public health:**  Leadership (convened large community gathering, etc.), program implementation, financial and technical support **Public health individuals involved:**  Program manager | Steps to Health King County was 1 of 40 community-level initiatives funded in 2003 as part of the Steps to a Healthier US initiative. Multiple interventions within eight projects were reported by level of intensity:  High intensity: one-on-one case management programs, including care coordination, community-based health education, physical activity for youth and seniors Medium intensity: multi-session programs or intensive one-time training or education, for community members, parents, and child care providers Low intensity: single-session or group education programs, school system and policy change, bicycle safety and promotion, youth health education | Program outcomes related to primary care provision, emergency department utilization, diabetes control, asthma management, health knowledge, and behaviour change were all statistically significant (p < .05). Participation in policy advocacy was limited, and staff reported being too busy with operational priorities to focus on advocacy.  Organizational level and policy changes were also reported. Primary reasons for lack of program integration and policy change included lack of time, focus on individual program activities, and perception of low payoff from collaborative activities. | None | **Initiation:** None **Implementation:** Developed place-based strategic plan; held senior leadership forum to implement system changes; created sector-specific health promotion strategies; created leadership and evaluation teams; hired staff members to increase coordination among organizations | Health services, physical and social environments | Midstream and downstream | Mixed | **High intensity:** US $900/person **Medium intensity**: US$115–$175/person **Low intensity:** $7/person | |
| Collie-Akers et al. 2007^42^ | | Analyzing a community-based coalition’s efforts to reduce health disparities and the risk for chronic disease in Kansas City, Missouri | **Design:** Empirical case study **Follow up:** Participatory research during intervention **Years of data collected**: 5 | **Country:** United States **Setting**: Urban | **Sample:** Online documentation of instances of community or system change **Key informant interviews**: n = 12  **Area characteristics:** Income: 24% households below poverty line **Race/ethnicity:** 57% African Americans, 8.5% Hispanics | **Time frame:** 2000 to 2004 **Sectors**: Public health, primary health care (community health centres), non-governmental organizations (neighbourhood associations, faith organizations, other public and private organizations) **Patterns of relationship between sectors**: Coordination  **Activities and relationships:** Coalition of multiple partners formed by Missouri Primary Care Association, including University of Kansas Work Group for Community Health and Development as scientific partner. Strengths included engagement of diverse parts of the community. **Role of public health**: Coalition focused on two minority populations in accordance with findings from a Kanas City Health Department report.  Public health individuals involved: Not specified | Kansas City - Chronic Disease Coalition: The coalition initiated a program called Pick Six, in which coalition partners were asked to identify six community changes that they could implement. From October 2001 through December 2004, coalition partners were given sub-contracts to implement the community changes that they had identified in the action plan. The partners consisted of 5 community health centres, 24 neighbourhood associations, 24 faith organizations, and several other public and private organizations. The coalition focused on two minority populations at high risk for cardiovascular disease and diabetes mellitus: African Americans and Hispanics. | The coalition facilitated 321 community changes from October 2001 through December 2004. Of these changes, 75% were designed to reduce residents’ risk for both cardiovascular disease and diabetes. The most common strategy was to provide health-related information to or enhance the health-related skills of residents (38%). | None | **Initiation:** Two separate health assessments identified health disparities; contract and funding from national organization (Centres for Disease Control and Prevention).  **Implementation:** Logic model for planning five interrelated phases; development of a plan for reducing disparities; hiring of a project manager; hiring (and later departure) of a community mobilizer to help implement the action plan; use of annual sub-contracts; and availability of targeted resources to neighbourhood and faith organizations. | Social and physical environments | Midstream | Targeted | Not specified | |
| Fazel et al. 2009^37^ | | A school-based mental health intervention for refugee children: an exploratory study | **Design:** Cohort analytic **Length of follow-up:** Immediately after intervention **Years of data collected**: 1 | **Country:** United Kingdom **Setting:** School | **Sample:** *n* = 141 school-age children (intervention: *n* = 47; comparison: *n* = 94). Two age- and sex-matched controls (one from a group of non-refugee ethnic minority children and one from a group of white children) were selected for each of the 47 children in the intervention group. **Characteristics**:  Age: School-age (first school: 4–8 years; middle school: 9–12 years; secondary school: 13–19 years) Race/ethnicity: India, Pakistan, Asia, Bangladesh, Balkans, other Intervention and comparison groups: Refugee, non-refugee ethnic minority and white children | **Time frame:** Not specified **Sectors:** Public health, education Patterns of relationship between sectors: Coordination **Activities and relationships:** Not specified  **Role of public health:** Provision of mental health services **Public health individuals involved:** Mental health professionals | School-based mental health service to address psychological needs of refugee children. Refugee children were discussed by mental health team, and children at greatest risk were seen.  Weekly individual counselling, with number of sessions per child varying according to need (2 to 5 weekly counseling sessions for most of the school year). | At the end of the study period, refugee children continued to have significantly higher Strengths and Difficulties Questionnaire (SDQ) total scores (*F* [2, 138] = 4.7, *p* = .011), emotional symptom scores (*F* [2, 138] = 8.6, *p* < .001), and peer problem scores (*F* [2, 138] = 6.3, *p* = .002) than those in the control groups. Over the study period (pre- vs. post-treatment), the total SDQ score in all groups decreased significantly (F [1, 138] = 5.9, p = .016), with the greatest changes evident in the peer problems scale (F [1, 138] = 8.1, p = .005) and the hyperactivity scale (F [1, 138] = 3.9, p = .05).  Hyperactivity scores decreased significantly more in the refugee group than in the control groups (mean change –0.96 [SD = 2.40] vs. –0.10 [SD = 1.98]; t = 2.12, p = .037), with a suggestion of an effect in the emotional symptoms score (mean change –0.72 [SD = 2.63] vs. 0.03 [SD = 2.02]; t = 1.73, p = .088). | Re-examination of the data comparing the outcomes of the 11 refugee children “directly seen” by the service and the 36 refugee children in the “consultation only” group revealed an interaction between time and group in the SDQ total score (*F* [1, 45] = 5.3, *p* = .026) and in the peer problems scale (*F* [1, 45] = 10.9, *p* = .002), with those who had been directly seen having significantly higher scores on the peer problems scale at baseline (*F* [1, 45] = 5.3, *p* = .026) and showing relatively greater improvement over the study period.  There was a non-significant improvement over time in scores on the emotional symptoms scale for refugee children directly seen by the service. At baseline, “caseness” criteria were met by 15 (32%) of the refugee children, 4 (9%) of the ethnic minority children, and 9 (19%) of the white children. At follow-up, caseness criteria were met by 11 (23%) of the refugee children, 2 (4%) of the ethnic minority children, and 10 (21%) of the white children, with the difference in caseness between the refugee and other children being significant at baseline but not at follow-up. (“Caseness” was defined as the combination of raised symptoms [SDQ score ≥ 14] and high impact scores [≥ 2]). | **Initiation:** Not specified **Implementation**: Creation of team of mental health professionals | Health services | Downstream | Targeted | Not specified | |
| Findley et al. 2006 ^32^ | | Community-based strategies to reduce childhood immunization disparities | **Design:** Cohort **Follow up:** 1 year **Years of data collected:** 3 | **Country:** United States **Setting:** Community | **Sample:** n = 1,502 children aged 19 to 35 months **Characteristics:** Income: Low income (study site, Northern Manhattan, includes the communities of Harlem and Washington Heights, which are among the most disadvantaged in the city of New York and the nation, with almost two thirds of families having incomes 200% below the poverty level, and a third receiving an income supplement) Race/ethnicity: African American (n = 281), Latino (n = 1,221). Two out of every five residents (40%) in these communities were foreign-born, the majority from the Dominican Republic but also from West Africa and other Latin American countries | **Time frame:** 1999–2004 **Sectors:** Public health, primary health care, academia, non-governmental organizations (social services, housing advocacy organizations) **Pattern of relationships between sectors:** Integration **Activities and relationships:** The program was designed, implemented, and directed by a large coalition. Activities were integrated into ongoing programs of community organizations. Staff were trained to provide immunization education and support.  **Role of public health:** Part of coalition that designed, implemented, and directed the intervention **Public health individuals involved:** Academic public health | Start Right was a community-based immunization promotion program of outreach and tracking for children younger than 5 years in Northern Manhattan, which was designed, implemented, and directed by a coalition of 23 community organizations. The program consisted of health education, reminders, follow-up, and incentives, all delivered in the context of programs routinely offered by coalition members. | Disparity reduction was assessed by comparing coalition immunization coverage rates for the 4:3:1:3:3 series (4 diphtheria-tetanus, 3-polio, 1 measles-mumps, rubella, 3 *Haemophilus influenza* b, and 3 Hepatitis B) with 2003 rates as determined by the National Immunization Survey. Coverage increased from 46.0% at enrollment to 80.5% at follow-up, matching nationwide rates for all children (*t* = 0.87) and for white children (*t* = 1.99). Immunization coverage for African American children in the study was greater than for African American children nationwide (78% *(SD=4.7)* v. 73.3% *(SD=3.2)*) (*t* = 2.90). Similarly, coverage for Latino children in the study was higher than for Latino children city and nationwide (83.7% *(SD=4.9)* v. 73.7% *(SD=9.5)* and 77.0% *(SD=2.1)*) (*t* = 2.32). Embedding immunization promotion into existing community immunization programs was successful in eliminating disparities in immunization rates. The most effective programs were those with direct linkages to health care systems targeting young children. | Age at enrollment and gender did not have a significant influence on immunization status  Age at enrollment (days): Adjusted Odds Ratio (AOR) = 1.10, CI [1,1] Gender: (male, female) AOR = 1.10, CI [.87, 1.39] Ethnicity: (Latino, other) AOR = 1.56, CI [1.14, 2.13] | **Initiation:** Followed an epidemic, at which time reports described low vaccination rates and inequities; 2 years of planning and piloting; built on existing programs and structures; coalition created **Implementation:** Shared accountability among partners; community ownership of program | Health services | Downstream | Targeted | No information | |
| Freeman et al. 2001^28^ | | Addressing children’s oral health inequalities in Northern Ireland: a research-practice-community partnership initiative | **Design:** Controlled clinical trial Follow-up: 1 year Years of data collected: 2 | **Country:** United Kingdom (Northern Ireland) **Setting:** School | **Sample**: *n* = 364 students in 16 schools (8 rural and 8 urban) eligible Year 1: (intervention: n = 118 students; control: n = 120 students) Year 2: (intervention: n = 99 students; control: n = 102 students) **Characteristics:** Age: 9 years old at baseline **Other**: Socioeconomically disadvantaged region | **Time frame:** Not specified **Sectors**: Public health, education, primary health care (specialized care: visiting ophthalmology service) **Patterns of relationships between sectors**: Coordination **Activities and relationships:** Policy developed by a team (dieticians, school meal advisors, teachers, health promotion officers, and local suppliers of school milk). Community-based practitioners negotiated and developed strategy with parents, teachers, and school governors. Research and Development Office of the UK Department of Health financed the evaluation.  **Role of public health**: Members of policy team  **Public health individuals involved**: Health promotion officer, dieticians, community-based practitioners | Boost Better Breaks: Each participating school or pre-school group had to have a written policy, approved by its board of governors, permitting the consumption of only milk and/or fruit at break time. Schools had to agree not to sell snacks high in fat or sugar in the school setting, and teachers had to agree not to reward students with candy.  Control: No written policy | Intervention group (low SES) had a mean DMFT (total number of decayed, missing due to caries and filled teeth) score of 1.58, CI [1.28, 1.89] compared to control group (high SES) mean score of 0.065, CI [0.38, 0.93].  The DMFT of intervention group (n = 99) year 1 was 1.13, CI [0.85, 1.40], compared to 1.58, CI [1.28, 1.89] in year 2.  Number of filled permanent teeth among intervention group changed from 0.49, CI [0.20, 0.77] in year 1 to 1.05, CI [0.69, 1.14] in year 2.  Program had a positive effect in terms of increasing the mean number of sound teeth among children attending schools in areas where socio-economic conditions were poor. | None | **Initiation:** The Boost Better Breaks break-time policy was based on the belief that using community development to negotiate and develop a break-time snacking policy within the school environment would help empower children to make the “healthy choice the easy choice.” The policy was developed by a team of dieticians, school meal advisors, teachers, health promotion officers, and local suppliers of school milk. **Implementation:** Multi-sectoral policy committee | Social and physical environments | Midstream | Targeted | Not specified | |
| Hollar et al. 2010^30^ | | Effective multi-level, multi-sector, school-based obesity prevention programming improves weight, blood pressure, and academic performance, especially among low-income, minority children | **Design:** Controlled clinical trial **Length of follow-up**: Fall 2004, Spring 2005, Fall 2005, Spring 2006 **Years of data collected:** 2 | **Country:** United States **Setting:** School | **Sample:** 4 intervention schools (n = 3,032 students), 1 control school (n = 737 students) Subgroup in free or reduced-cost lunch program: n = 1,197 **Characteristics:** Age: mean 8 years, range 4–13 Sex ratio: 51% female, 49% male Race/ethnicity: 50.2% Hispanic, 33.4% White, 8.0% Black, 8.4% other (multi-ethnic, Asian, American Indian) | **Time frame:** 2004 to 2006 **Sectors:** Academia, education (school administration and cafeteria), district food services, district wellness committee, Department of Agriculture Food and Nutrition Service, and media (i.e., magazine) **Patterns of relationships between sectors:** Cooperation, coordination **Activities and relationships:** Not specified **Role of public health**: Not specified **Public health individuals involved:** Dietitian, district wellness committee members | Healthier Options for Public Schoolchildren (HOPS)/OrganWise Guys (OWG): an elementary school–based obesity prevention intervention designed to keep children at a normal healthy weight and to improve health status and academic achievement. Components included dietary intervention, curriculum, and physical activity. Free or reduced-cost lunch program provided to children from low-income families. | Children in intervention group experienced greater decrease in body mass index (BMI) percentile than children in control group in year 1; difference between improvements in BMI percentiles reached statistical significance in year 2 (*p* = .007). Females in the control group school experienced significant increase in mean systolic blood pressure (from 98.37 to 101.44 mm Hg) (p < .001); males in both groups had significant increases in systolic blood pressure during summer (from 100.83 to 101.94 mm Hg in intervention group, from 99.28 to 101.93 mm Hg in control group) (p < .0001). Increases in diastolic blood pressure were seen during summer in both sexes and in both the intervention and control groups (p < .0001). | In the sub-sample of children receiving free or reduced-cost lunches, mean BMI (in terms of z score) changed from 0.61 (standard deviation [SD] = 1.19) at baseline to 0.71 (SD = 1.09) at final measurement in the intervention group and from 0.98 (SD = 0.88) at baseline to 1.05 (SD = 0.85) at final measurement in the control group (p = .0013). Also in the sub-sample of children receiving free or reduced-cost lunches, mean weight (in terms of z score) changed from 0.61 (SD = 1.14) at baseline to 0.65 (SD = 1.12) at final measurement in the intervention group and from 0.90 (SD = 0.98) at baseline to 0.95 (SD = 1.00) at final measurement in the control group (p = .011).  Hispanic and white children in the intervention schools were significantly more likely to have higher Florida Comprehensive Achievement Test math scores than their counterparts in the control school (p < .001). | **Initiation:** None described **Implementation:** None described | Physical and social environments, food security | Midstream | Mixed | Not specified | |
| Jackson et al. 2011^31^ | | Reduced acute hospitalization with the healthy housing programme | **Design:** Interrupted time series **Follow-up:** 2.3 years **Years of data collected:** 10 | **Country:** New Zealand **Setting:** Community-based, district level | **Sample:** n = 9,736 residents of 3,410 homes **Characteristics:** Age: 0–4 years of age, 24.75%; 5–34 years of age, 50.6%; 35 years or older, 24.5% | **Time frame:** July 1999 to January 2009  **Sectors:** Public health, housing  **Patterns of relationship between sectors:** Integration **Activities and relationships:** Joint initiative between housing and health boards. Assessment undertaken by housing area coordinator and public health nurse. **Role of public health:** Co-lead in the joint housing initiative, part of the assessment team **Public health individuals involved:** Public health nurse | Healthy Housing Programme, a joint initiative between Housing New Zealand Corporation and Counties Manukau, Auckland, Hutt Valley, and Northland District Health Boards with three related dimensions: health, housing, and social. The study investigated the impact of housing modifications to reduce overcrowding; insulation and ventilation improvements; and health and social service assessments, referrals, and linkages for acute admission to hospital. | People aged 5–34 years had fewer acute admissions to hospital after the intervention than before (hazard ratio [HR] 0.77, confidence interval [CI] [0.70, 0.85]). For children aged 0-4 years, the HR was 0.89, CI [0.79, 0.99]. Among adults 35 years of age or older, there was a non-significant increase. When the causes of hospital admission were restricted to those related to housing, a further decline in HR was seen: 0.88, CI [0.74, 1.05], for those 0–4 years old, 0.73, CI [0.58, 0.91] for those 5–34 years of age and 1.31, CI [1.09, 1.56] for those 35 years of age or older. | None | **Initiation**: Not specified **Implementation**: Not specified | Housing | Midstream | Targeted | Not specified | |
| Macnab et al. 2008^41^ | | 3-year results of a collaborative school-based oral health program in a remote First Nations community | **Design:** Cohort **Follow-up**: 3 years **Years of data collected:** 3 | **Country:** Canada **Setting:** School | **Sample:** *n* = 58 children enrolled, with 26 children receiving the complete intervention **Characteristics**:  Age: Kindergarten to grade 10 at the outset, subsequently to grade 12 Race/ethnicity: Aboriginal | **Time frame:** Not specified  **Sectors:** Public health, primary health care, academia, education, non-governmental organization **Pattern of relationships between sectors:** Coordination **Activities and relationships**: Initiated by University of British Columbia (UBC) Pediatric Residency Program. Meeting with community elders and community health staff identified health problem. Option of school-based intervention was selected, and school principal and teachers were involved in design of program. UBC team implemented the intervention, working with nurses, the school, and band council to maintain the program.  **Role of public health**: Involved in meeting that identified health problem **Public health individuals involved:** Public health nurse | School-based program to improve knowledge and practices related to oral health, using brush-ins and application of topical fluoride varnish and/or rinses, dental health anticipatory guidance by the pediatric residents during well-baby and well-child visits, and classroom presentations by the pediatric residents about a variety of health topics, including oral health. | Before the intervention, 8% of the children were cavity-free (based on assessment of 45% of the 58 children). Following 3-year intervention, 32% were cavity-free. Among the 13 children assessed both pre- and post-intervention, dmfs/DMFS improved significantly (p < .005). The visiting hygienist noted increased knowledge about oral health.  The community was able to see a marked improvement and remained very positive about the program. | None | **Initiation:** Meetings to identify health problem, appropriate response, and program design. **Implementation:** Not specified | Health services | Downstream | Targeted | Not specified | |
| Melvin 2006 ^38^ | | A collaborative community-based oral care program for school-age children | **Design:** Cohort **Follow-up**: Immediately after intervention **Years of data collected:** 4 | **Country:** United States **Setting:** School | **Sample:** Children in three district schools, year 1 (n = 1, 144), year 2 (n = 353), year 3 (n = 635) **Characteristics:** Age: School-age (grades 1 to 6) Other criteria: Low income, refugees | **Time frame:** 2001 to 2005 **Sectors:** Public health, primary health care, education, academia (nursing faculty) **Patterns of relationships between sectors:** Coordination, integration  **Activities and relationships**: A community nurse specialist brought issue to university nursing faculty and Vermont Department of Health. A Dental Access Committee was created, with program delivery funded by health department and local hospital. Dental hygienist performed visual examination and provided referrals to a dentist. **Role of public health:** Members of Dental Access Committee  **Public health individuals involved:** Public health nurse, executive and senior management | Tooth Tutor Program, aimed at children in grades 1-6, placed most of its emphasis on identifying children without a “dental home” and on increasing the number of children receiving oral preventive services and routine care in a dental office. Dental hygienists within schools identified children in need of services (by visual screening) and set up appointments with community dentists for the provision of preventive, restorative, and emergency dental care. The classroom component included yearly presentations. | Phase 1 (years 1 and 2): In September 2001 (year 1), 59% of children in the three schools had a dental home, and by June 2002, 78% of children had a dental home. In September 2002 (year 2), 51% of children had a dental home, and by June 2003, 87% of children had a dental home.The program served 1, 144 children in year 1 and 353 children in year 2. In year 1, 75 children (52% of the target group) received preventive care and 32 (22%) received restorative care. In year 2, 212 children (60%) received preventive care, and 39 (11%) received restorative care. Phase 2 (years 3 and 4): As of June 2004, a total of 635 children had been seen by community dentists. Services provided included cleaning, application of sealants, fluoride treatments, radiography, placement of crowns, extractions, and oral surgery.  In fall 2004, a school-based dental clinic was opened in one of the original three schools, so that dental care could be provided on site. During that year, 212 students were seen on site.  The breakdown of care provided in phase 2 was 54% diagnostic or preventive services and 46% restorative services. The increase in the need for restorative services in years 3 and 4 was attributable to continued immigration of refugee children requiring extensive dental care. Another contributing factor may have been that new children continued to move into the school district. | None | **Initiation:** Champion, multi-sectoral committee **Implementation:** Multi-sectoral committee, funding | Health services, social and physical environments | Downstream | Mixed | $70 per child served | |
| Metzel et al. 2005^43^ | | State-level interagency agreements for supported employment of people with disabilities | **Design:** Qualitative descriptive **Data collection**: May 1999 to January 2000 | **Country:** United States **Setting:** State level | **Sample:  Document review:** Agreements from six states, focusing on mental illness [n = 2], most severe disabilities [n = 2], developmental disabilities [n = 1], and transitioning studies [n = 1]. Number of partner agencies: two (n = 4), four + two guests (n = 1), or seven (n = 1). **Key information interviews:** n = 20 | **Time frame:** Not specified **Sectors:** Public health (Department of Mental Health), education, employment and labour  **Patterns of relationships between sectors:** Cooperation, coordination  **Activities and relationships:** written interagency agreements using language that promoted cooperation and outlined roles and responsibilities of partners. **Role of public health:** Not specified **Public health individuals involved:** Middle managers | The study investigated six interagency agreements for supported employment to identify the conditions and qualities necessary to increase the number of people in supported employment.  To determine the effect of written interagency agreements outlining planned coordination for supported employment, researchers asked the following questions: 1. What is necessary for the development of a potentially good interagency agreement for supported employment?  2. What is necessary for the implementation of a potentially good interagency agreement? 3. What are the positive outcomes of the interagency agreements for supported employment? | Increase in supported employment: Five states succeeded in supporting more people with disabilities in employment. Estimates indicated a 25% yearly increase in employment from 1994 to 1999. In 1997 there was an increase of 30%, with 200–300 young people benefiting from vocational assessment and employment opportunities, and between 1995 and 1996 there was an increase of 14%. Representatives from three states also mentioned increased visibility of supported employment.  Increase in coordination and collaboration: Representatives from three states described increased coordination and cooperation (e.g., by altering processes, changing systems, coordinating budgets). | None | **Initiation:** Legislation requiring inter-agency cooperation and coordination at the state level. Funding, fulfillment of legal mandates, formal endorsement of supported employment, mission or vision statements. **Implementation**: Written interagency agreements, which included population-specific agreements, resource commitments, partners’ roles and responsibilities, and expected outcomes. Themes of good collaboration included champions, strong working relationship across agencies and team members, common language, regular and frequent meetings. | Employment | Upstream | Targeted | Not specified | |
| Pechter et al. 2009^36^ | | Reducing hazardous cleaning product use: a collaborative effort | **Design:** Case study **Length of follow-up:** Not specified **Years of data collected:** 1 | **Country:** United States **Setting**: Workplace | **Sample:** *n* = 140 workers **Characteristics:** Low-income workers, immigrant workers | **Timeframe:** 2005 **Sectors**: public health, employment and labour (union), non-governmental organization (NGO) (Massachusetts Coalition for Occupation Safety and Health) **Pattern of relationships between sectors:** Coordination  Activities and relationships: Union responded to worker concerns. NGO developed survey and supported surveillance of cleaning products used. By invitation, Occupational Health Surveillance Program of the Massachusetts Department of Public Health provided technical information about health effects and preventive measures. **Role of public health**: Translation of surveillance data into practical recommendations for immediate change.  **Public health individuals involved**: Not specified | Multi-year project led by immigrant cleaning workers with their union, Service Employees International Union, Local 615, and support from Massachusetts Coalition for Occupational Safety and Health to address exposure to hazardous chemicals. | Development of a workplace policy calling for elimination of the most hazardous chemicals, reduction in the number of products used, ban on mixing products, and improvements in safety training. | None | **Initiation:** Union leadership, participatory approach **Implementation:** Staff, survey, reports | Employment and working conditions | Midstream | Targeted | Not specified | |
| Peifer & Perez 2011^39^ | | Effectiveness of a coordinated community effort to promote early literacy behaviors | **Design:** Interrupted time series  **Follow-up**: 2 years  **Years of data collected:** 2 | **Country:** United States  **Setting**: Community | **Sample:** Intervention delivered to approximately 1,500 families, surveys of two independent samples collected in 2001 (n = 300 parents) and 2003 (n = 216 parents)  **Characteristics:**  Annual income: Survey I, 47.7% with less than $15,000, 48.1% with greater than $15,000 but less than $30,000, 4.1% with greater than $30,000 but less than $50,000; survey II (post-intervention), 25.5% with less than $15,000, 43.0% with greater than $15,000 but less than $30,000, 31.5% with greater than $30,000 but less than $50,000  Race/ethnicity: Survey I, 82.6% Hispanic; survey II, 69.4% Hispanic Education level less than high school: Survey I, 83 (32.2%), survey II, 84 (38.9%) | **Time frame**: Interventions began in 1998; data were collected in 2001 and 2003 **Sectors:** Public health, primary health care, non-governmental organizations, public library **Pattern of relationships between sectors**: Cooperation  **Activities and relationships**: Four separate community programs; coordination to deliver and evaluate the intervention (providing access to books to target population). Interventions built on existing programs (home visits, clinic visits, child care centres) and involved partnering with public library system. **Role of public health**: Part of intervention delivery team  **Public health individuals involved:** Public health nurse | Four coordinated programs: Prenatal to Three initiative, “Raising a Reader,” Reach Out and Read, California’s FIRST 5 Commission. The four programs had common elements, specifically book distribution programs based in clinical settings, child care centres, and home visitation programs. The intent of the programs was to communicate the message that reading to infants and young children and accessing services at the public library are beneficial. | Data comparison between the two time periods showed the following changes (data reported as ratio between proportions [%], 2003/2001):  · 77% increase in parents reporting that they showed books to their infants on a daily basis  ·61.44% increase in parents reading aloud to their infants on a daily basis  ·89.29% increase in parents playing with child  ·52.96% increase in parents drawing pictures with child | None | **Initiation:** Building on existing programs and relationships; funding  **Implementation:** Not specified | Early childhood development | Midstream | Targeted | Not specified | |
| Sherring et al. 2010^35^ | | A working reality: evaluating enhanced intersectoral links in supported employment for people with psychiatric disabilities | **Design:** Cohort  **Follow-up**: 2 years  **Years of data collected**: 2 | **Country:** Australia **Setting:** Regional level, metropolitan, regional, rural, and remote communities | **Sample:** *n* = 43 people with mental illness  **Sex ratio**: 79% male (n = 34), 21% female (n = 9) **Education:** 46.5% high school (n = 20), 7% bachelor’s degree (n = 3) **Other criteria**: Program targeted young people in the first 5 years of illness. | **Time frame:** September 2006 to September 2008 **Sectors**: Public health, primary health care, employment and labour **Patterns of relationships between sectors**: Information sharing, integration  **Activities and relationships:** Program staffed by occupational therapists and an employment consultant. Intersectoral approach consisted of integration of occupational therapists with community mental health and employment services. Participating staff agreed to principles and established formal communication process. Funded by Department of Education, Employment and Workplace Relations. Implementation by occupational therapist, who assessed personal needs, assisted with access to employment services, and provided clinical support. Employment consultant responsible for all job search activities. **Role of public health:** Providing mental health services, performing evaluation of program **Public health individuals involved**: Health promoter | Vocational Education, Training and Employment (VETE) demonstration project: supported employment program in which formal links were created between a community mental health team and three employment services to evaluate the application of evidence-based employment programs in the Australian context. | Participants who held a job in the year preceding entry into the VETE program were employed for a greater proportion of the total participation period (mean 64.4%, *SD* 28.3) than those who had not held a job before entry into the program (mean 36.6%, *SD* = 27.6) (*df* = 31, *t* = 2.858, *p* < .01). Total BPRS at baseline was significantly lower among participants who got a job (mean 34.2, SD = 6.9) than among those who did not get a job (mean 39.8, SD = 6.6) (df = 41, t = 2.28, p < .05). Total score on Work-related Self-efficacy Scale was significantly higher among those who obtained employment (mean 80.5, SD = 12.3) than among those who did not get a job (mean 68.7, SD = 11.46) (df = 39, t = 2.68, p < .01). No significant differences for any other predictors. | None | **Initiation:** Both formal (monthly case reviews, regular joint appointments) and informal (phone and email) communication processes established. Clearly defined roles established during early stages of the partnership. **Implementation:** Staff, funding, education, communication between sectors, culture change | Employment and working conditions | Midstream | Targeted | Not specified | |
| Smith et al. 2009^27^ | | Partners in health? A systematic review of the impact of organizational partnerships on public health outcomes in England between 1997 and 2008 | **Design:** Systematic review **Data sources:** 18 electronic databases covering academic research, local and central government studies, and grey literature in the medical, social sciences, and economic literatures (January 1997 to June 2008); bibliographies of identified articles were also searched | **Country:** England | **Sample:** 15 studies, relating to six different interventions, met review criteria: specifically, provided data on impact of partnerships on public health outcomes (improvement in health and/or reduction in health inequalities), either directly (e.g., effects of partnerships or of partnership-implemented interventions on self-reported health) or indirectly (e.g., by raising the policy profile of health inequalities). |  | a) Health Action Zones (HAZs): area-based initiatives intended to develop partnerships involving the National Health Service (NHS), local government, and other sectors, with the aim of tackling ill health and persistent inequalities in the most disadvantaged communities across the UK. The initiatives aimed to address social and economic determinants (e.g., services providing advice on benefit support), promote healthy lifestyles (e.g., smoking cessation services), empower individuals and communities (e.g., “Stepping Out,” a program of leisure and sports activities for people with physical and sensory disabilities), and improve health and social care services (e.g., Integrated Substance Misuse Service). HAZs were launched in 1998 and received a total of £320 million over a 3-year period.  b) Health Improvement Programmes (HImPs): action plans developed by NHS and local government bodies and introduced in 1999; renamed Health Improvement and Modernisation Plans in 2001. The plans set out how these organizations (with volunteer and private sector input where deemed appropriate) intended to improve the health of local populations and reduce health inequalities. The programs offered a 3-year plan for identifying local health needs and developing relevant strategies to improve health and health care services at the local level. HImPs were founded on the basis of multi-agency partnerships between local government and strategic health authorities. c)  New Deal for Communities (NDC): part of the Neighbourhood Renewal Strategy, developed to tackle health and social inequalities experienced by the 39 most deprived communities in the UK. In partnership with local communities, the NDC sought to address embedded issues of deprivation and long-term poverty by improving outcomes in terms of housing, education, employment, and health. Interventions focused mainly on promoting healthy lifestyles, enhancing service provision, developing the health workforce, and working with young people.  d) Health Education Authority Integrated Purchasing Programme: developed by the Health Education Authority and operational between 1996 and 1999. The overall aim was to support partnerships between local authorities, primary care groups, and health authorities in improving health. Five demonstration projects were launched, each involving a local partnership to tackle health inequalities. Other elements of the program included a national Practice Exchange Network, a learning and dissemination program, and a knowledge resource base.  e) Healthy Living Centres (HLCs): introduced in 1998 to tackle the broader determinants of health inequalities and to improve health and well-being at the local level. Funding was awarded for 352 community projects, which varied in terms of their focus, ranging from service-related issues to activities addressing unemployment, poverty, and social exclusion. Working in partnership was presented as an underpinning concept of HLCs. The interventions included health-focused projects such as a physical activity outreach program in rural communities; support programs such as the Community Health Information Project, which trained members of the local community to act as ambassadors for the HLCs; and services such as “bumps to babies,” which provided midwifery and health visiting services for young families. Although some HLCs were still in existence at the time the systematic review was performed, a lack of clarity about funding meant that the future of many HLCs was unclear.  f) National Healthy School Standard: led by a partnership between the Department of Health, the Department for Education and Skills, and the Health Development Agency. Its three key objectives were to raise pupil achievement, to promote social inclusion, and to contribute to reducing health inequalities. | Four of the 15 studies included a quantitative element and produced a mixed picture of the impacts of partnerships. Qualitative studies suggested that some partnerships increased the profile of health inequalities on local policy agendas. The variation in design of the partnership interventions and of their evaluations made it difficult to assess whether the observed impacts resulted from the partnerships. |  |  | Interventions addressed a range of determinants of health such as social exclusion, income, employment, housing, and poverty. |  | Targeted |  | |
| Wills et al. 2010^29^ | | Improving school readiness with the Before School Check: early experience in Hawke’s Bay | **Design:** Cohort  **Follow-up:** 1 month after intervention and each month thereafter for 10 months  **Years of data collected**: 1 | **Country:** New Zealand **Setting**: Health district (largely rural population) | **Sample:** All preschool children in Hawke’s Bay. In 2006, there were 34,101 children and approximately 2,200 deliveries in the region, with 56% of babies born being categorized in New Zealand Deprivation Index deciles 8-10. | **Time frame:** 2009 **Sectors:** Public health; primary health and non-governmental organizations (social services)  **Pattern of relationships between sectors**: Cooperation  **Activities and relationships:** Hawke's Bay District Health Board used a formal tendering process to select the program provider (Hawke's Bay Primary Health Organization). Clinical advisory groups (with numerous representatives from health, education, and social services) were formed and were described as critical to success of the program (through engagement and commitment of stakeholders). Training was delivered by public health personal, primary health care providers, academia, and consultants. Program was promoted by public health and health staff within existing service delivery. Data collection and evaluation were performed by Hawke's Bay Primary Health Organization.  **Role of public health:** Leadership for other stakeholders, training of nurses to perform screening, active promotion of the program within existing service delivery, creation of clinical advisory group  **Public health individuals involved:** Public health nurse, program manager | Before-School Check: screening program for school readiness, including child health questionnaire, vision, hearing, and oral health screening, measurement of height and weight, assessments of behaviour (with the Strength and Difficulties Questionnaire) and of development (with the Parent Evaluation of Developmental Status), health promotion and education, and referrals as indicated. | At baseline: Data reported in graph format; checks and referrals appear to be below 10%.  Estimated effect of program: 1,848 checks (84% of cohort) completed over the 10-month intervention period; 50% referral rate maintained. | Income quintile 1: 110% screened Income quintile 2: 90% screened Income quintile 3: 90% screened Income quintile 4: 80% screened Income quintile 5: 75% screened | **Initiation:** Based on piloted programs  **Implementation:** Clinical advisory group, promotion of activities within existing programs | Early childhood development, health services | Downstream | Mixed | Not specified | |
